# Biochemical and Proteomic Analyses in Drought-Tolerant Wheat Mutants Obtained by Gamma Irradiation

**DOI:** 10.3390/plants13192702

**Published:** 2024-09-27

**Authors:** Ayşe Şen, Tamer Gümüş, Aslıhan Temel, İrfan Öztürk, Özge Çelik

**Affiliations:** 1 Department of Biology, Faculty of Science, Istanbul University, Istanbul 34134, Türkiye; 2 Department of Molecular Biology and Genetics, Faculty of Science and Letters, Istanbul Kultur University, Istanbul 34156, Türkiye; t.gumus@iku.edu.tr (T.G.); ocelik@iku.edu.tr (Ö.Ç.); 3 Department of Molecular Biology and Genetics, Faculty of Science, Istanbul University, Istanbul 34134, Türkiye; atemel@istanbul.edu.tr; 4 Trakya Directorate of the Institute of Agricultural Research, Edirne 22030, Türkiye; irfan.ozturk@tarim.gov.tr

**Keywords:** *Triticum aestivum* L., drought tolerance, proteomics, chloroplast proteins, mitochondrial proteins, pyruvate dehydrogenase complex, rubisco, guaiacol peroxidase, homeostatic process

## Abstract

The bread wheat cultivar (*Triticum aestivum* L. cv. Sagittario) as a parental line and its mutant, drought-tolerant lines (Mutant lines 4 and 5) were subjected to polyethylene glycol (PEG)-induced drought. Drought stress resulted in decreased chlorophyll levels and the accumulation of proline and TBARS, despite increases in activities of catalase, peroxidase, and superoxide dismutase enzymes. Transcription of the genes encoding these enzymes and delta-1-pyrroline 5-carboxylase synthetase was induced by drought. 2-DE gel electrophoresis analysis identified differentially expressed proteins (DEPs) in the mutant lines, which are distinguished by “chloroplast”, “mitochondrion”, “pyruvate dehydrogenase complex”, and “homeostatic process” terms. The drought tolerance of the mutant lines might be attributed to improved photosynthesis, efficient ATP synthesis, and modified antioxidant capacity. In addition to proteomics data, the drought tolerance of wheat genotypes might also be assessed by chlorophyll content and *TaPOX* gene expression. To our knowledge, this is the first proteomic analysis of gamma-induced mutants of bread wheat. These findings are expected to be utilized in plant breeding studies.

## 1. Introduction

Drought is one of the main limiting factors for plant production and can be caused by limited uptake of water, high soil salinity, ground frost, or increasing transpiration rates during hot seasons [1,2,3]. Plants take up enormous amounts of water to maintain high water content in their tissues [1,3]. Even low levels of drought can impair growth, not only due to a decline in turgor but also because of the reduced photosynthesis resulting from the accumulation of abscisic acid (ABA), of which levels increase upon perception of drought and compensate the effects of drought conditions by inhibiting stomatal conductance, stimulating root cell elongation, and regulating the expression of stress-responsive genes [4,5,6]. Stomatal closure restricts not only water loss but also CO_2_ uptake from the atmosphere [1]. At a certain point, when cytoplasm does not contain enough water to maintain normal functions of biomolecules, cytoplasm becomes more viscous and crowded. Under such conditions, increased interactions between biomolecules result in aggregation that might be irreversible [7]. When persistent drought stress occurs, it can compromise membrane integrity and cell viability [1]. Therefore, it is necessary to produce plants that can use water efficiently and improve agricultural applications due to climate change and the increasing global population. Elucidating the mechanisms involved in drought response or tolerance is a prerequisite for breeding approaches [1,2,3].

One of the important effects of drought stress in plants is the increase in the production of reactive oxygen species (ROS) in the cell. Increased free radical production triggers oxidative stress and damages the important structural components of the cell, such as DNA, protein, chlorophyll, and cell membranes. If plants cannot cope with this stress, a process that leads to the death of the living being is triggered. Antioxidant metabolism, consisting of enzymatic and non-enzymatic antioxidant agents, plays a vital role in neutralizing ROS formed due to salt stress. Drought tolerance is positively associated with antioxidant enzyme activity, e.g., catalase (CAT: EC 1.11.1.6), superoxide dismutase (SOD: EC 1.15.1.1), and guaiacol peroxidase (POX: EC 1.11.1.7), and with the accumulation of non-enzymatic antioxidant compounds [8]. Accumulation of proline, a compatible osmolyte species, is a common response to drought stress and may help alleviate the negative effects of this stress. In addition to providing tolerance to drought stress, accumulation of proline during stress may also help as a source of organic nitrogen for the plant during recovery from stress [9].

The genetic methods for the development of stress tolerance in plants can be achieved by several mechanisms, including generation of transgenic and edited plants [10] and mutation breeding. Genetic engineering involves the introduction of a foreign gene or manipulation of the expression levels of native genes [11]. In genetic engineering, expression of the introduced gene can also be optimized [12]. Abiotic stress tolerance through genetic engineering has been quite successful, however, rather confined to approaches using single genes. Genome editing techniques, e.g., clustered regularly interspaced short palindromic repeat (CRISPR)/CRISPR-associated protein 9 (Cas9), offer highly efficient opportunities to increase abiotic stress tolerance. Using this precise and robust method, not only tolerance genes are targeted but also sensitivity genes, which may interfere with tolerance genes, can be manipulated [13]. Nevertheless, evaluation of transgenic plants and the legal procedures for their cultivation and commercialization remain challenging [10,12].

Another genetic approach for the development of stress-tolerant plants is mutation breeding. Physical and chemical mutagens enhance the rate of mutation and, as a result, genetic variability [14]. The main aim of the mutation breeding is to improve well-adapted varieties through altering 1–2 traits, e.g., stress resistance or crop quality [15]. Radiation mutagenesis can induce more complex mutations than chemical mutagenesis and has been used for the improvement of crops for almost 100 years. One of the physical mutagens is gamma ray and has been effective in plant breeding as they induce point mutations and small deletions [14]. Because radiation mutagenesis is capable of introducing a wide mutation spectrum, it is still indispensable for the development of improved genotypes [16]. A total of 112 mutant varieties tolerant to drought are currently listed in the FAO-IAEA mutant variety database, and 33 of them belong to *T. aestivum* [17]. Polyethylene glycol (PEG) is used to induce osmotic stress and select drought-tolerant plants, which can be characterized according to their proline and ABA contents, their antioxidant enzyme activity [14,18,19], and their molecular [20] and Raman spectroscopy profiles [21].

Under drought stress, gene expression undergoes extensive reprogramming. The genes that respond to drought can include transcription factors, proteins involved in water transport, enzymes responsible for producing and detoxifying ROS, enzymes involved in synthesizing specific solutes and sugars, and chaperones [1]. Molecular mechanisms of drought stress response occur also at post-transcriptional levels. For instance, farnesylation and ubiquitylation of proteins are required for protein-protein interactions or removal of existing proteins, which are parts of stress signaling and tolerance [1,22]. In addition to alteration of protein synthesis and modifications as a drought stress response, lack of water in the cytoplasm can directly result in loss of the 3D structure and aggregation of proteins [7]. The proteome is the entire set of proteins in a cell or an organism and is more complex and dynamic than the genome and transcriptome, as mRNA levels may not correlate with the protein levels and functions [23,24]. Therefore, proteomics became a powerful method for the identification of drought-responsive genes and proteins in plants. Drought stress proteins prevent damage to photosynthesis, photorespiration, and other physiological processes [24]. Differentially expressed proteins (DEPs) in wheat under drought stress are mainly involved in carbohydrate metabolism, detoxification, and regulation of gene expression. The levels of up- or down-regulation and changes differ among wheat genotypes that have contrasting drought tolerance [25].

The aim of this study was to comparatively investigate the drought tolerance of the commercial parental cultivar (*T. aestivum* cv. Sagittario) and its seventh generation mutant lines (4 and 5) induced by gamma irradiation. These mutant lines had been previously generated by our group [20]; they were reported to be genetically distinct from the parental line and exhibited better performance under drought conditions, depending on the fixed genome opening. For this purpose, immature embryos were first subjected to PEG-induced drought treatment under in vitro conditions. Then, physiological e.g., fresh weight (FW) and plantlet length (PL) and biochemical parameters e.g., chlorophyll, and thiobarbituric acid-reactive substances (TBARS) and proline contents as indicators of stress-derived damages, and superoxide dismutase (SOD, EC 1.15.1.1), catalase (CAT, EC 1.11.1.6), and peroxidase (POX, EC 1.11.1.7) activities were measured. Expression levels of the genes *TaCAT*, *TaP5CS*, *TaPOX*, and *TaSOD2* were also investigated. Up- or down-regulated proteins in either mutant lines or the parental line were clustered according to Gene Ontology (GO) terms. Stress tolerance indices (STIs) of the mutant lines were calculated based on the quantitative data. Proteome profiles of a bread wheat line and its mutant lines are presented for the first time in this study.

## 2. Results

### 2.1. Plant Growth

PEG treatment inhibited plant growth and less greenish leaf color in the parental line (Figure S1A), mutant line 4 (Figure S1B), and mutant line 5 (Figure S1C). PEG treatment significantly (*p* < 0.001) decreased, while genotype did not affect (*p* > 0.05) FW. There was no interaction (*p* > 0.05) between treatment and genotype for FW. PEG treatment caused the highest (67.12%) decrease in FW in the parental cultivar (Table S1).

PEG treatment significantly (*p* < 0.05) decreased while genotype did not affect (*p* > 0.05) PL. There was no interaction (*p* > 0.05) between treatment and genotype for PL. PEG treatment caused the highest (50.43%) decrease in PH in the parental cultivar (Table S2).

### 2.2. Chlorophyll, Proline, and TBARS Contents

**Figure 1:**
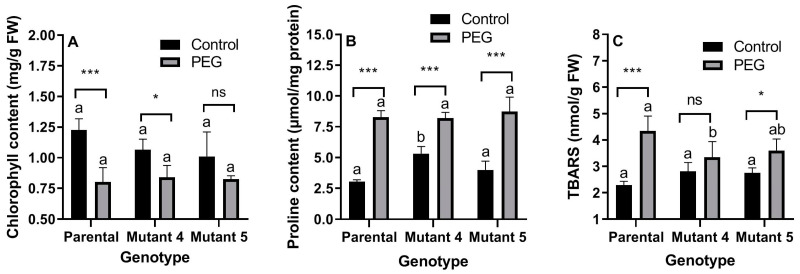
Effects of PEG-induced osmotic stress on chlorophyll content (**A**), proline content (**B**), and TBARS content (**C**) of the parental line (commercial cultivar) and its mutant lines 4 and 5. Values are the arithmetic means of biological pentaplicates (*n* = 5), and the error bars correspond to the standard deviations (SD). Significant differences were determined according to Tukey’s HSD test performed after a 2-way ANOVA. The letters on the columns represent the statistical significance among the genotypes under the same conditions (control or PEG). Columns indicated by different letters are statistically different (*p* < 0.05). Asterisks represent the statistical significance of a genotype between different conditions as follows: * *p* < 0.05, *** *p* < 0.001, and ns (not significant).

PEG treatment significantly (*p* < 0.01) decreased, while genotype did not affect (*p* > 0.05) chlorophyll content. There was no interaction (*p* > 0.05) between treatment and genotype for chlorophyll content. PEG treatment caused the highest (34.57%) decrease in chlorophyll content in the parental cultivar (Figure 1A).

PEG treatment significantly (*p* < 0.01) increased, and genotype significantly (*p* < 0.01) affected proline content. There was a highly significant interaction (*p* < 0.01) between treatment and genotype for proline content. PEG treatment caused the highest (171.60%) increase in proline content in the parental cultivar (Figure 1B).

PEG treatment significantly (*p* < 0.01) increased, while genotype did not affect (*p* > 0.05) TBARS content. There was a highly significant (*p* < 0.01) interaction between treatment and genotype for TBARS content. PEG treatment caused the highest (89.45%) increase in TBARS content in the parental cultivar (Figure 1C).

### 2.3. Antioxidant Enzyme Activity

**Figure 2:**
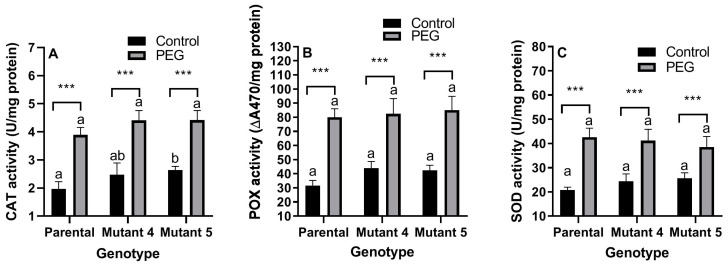
Effects of PEG-induced osmotic stress on the activities of the enzymes CAT (**A**), POX (**B**), and SOD (**C**) of the parental line (commercial cultivar) and its mutant lines 4 and 5. Values are the arithmetic means of biological pentaplicates (*n* = 5), and the error bars correspond to the standard deviations (SD). Significant differences were determined according to Tukey’s HSD test performed after a 2-way ANOVA. The letters on the columns represent the statistical significance among the genotypes under the same conditions (control or PEG). Columns indicated by different letters are statistically different (*p* < 0.05). Asterisks represent the statistical significance of a genotype between different conditions as follows: *** *p* < 0.001.

PEG treatment significantly (*p* < 0.01) increased and genotype significantly (*p* < 0.01) affected CAT activity. There was no interaction (*p* > 0.05) between treatment and genotype for CAT activity. PEG treatment caused the highest (97.96%) increase in CAT activity in the parental cultivar (Figure 2A).

PEG treatment significantly (*p* < 0.01) increased and genotype significantly (*p* < 0.05) affected POX activity. There was no (*p* > 0.05) interaction between treatment and genotype for POX activity. PEG treatment caused the highest (152.69%) increase in POX activity in the parental cultivar (Figure 2B).

PEG treatment significantly (*p* < 0.01) increased, while genotype did not affect (*p* > 0.05) SOD activity. There was a significant (*p* < 0.05) interaction between treatment and genotype for SOD activity. PEG treatment caused the highest (105.76%) increase in SOD activity in the parental cultivar (Figure 2C).

### 2.4. Changes in mRNA Transcript Levels

**Figure 3:**
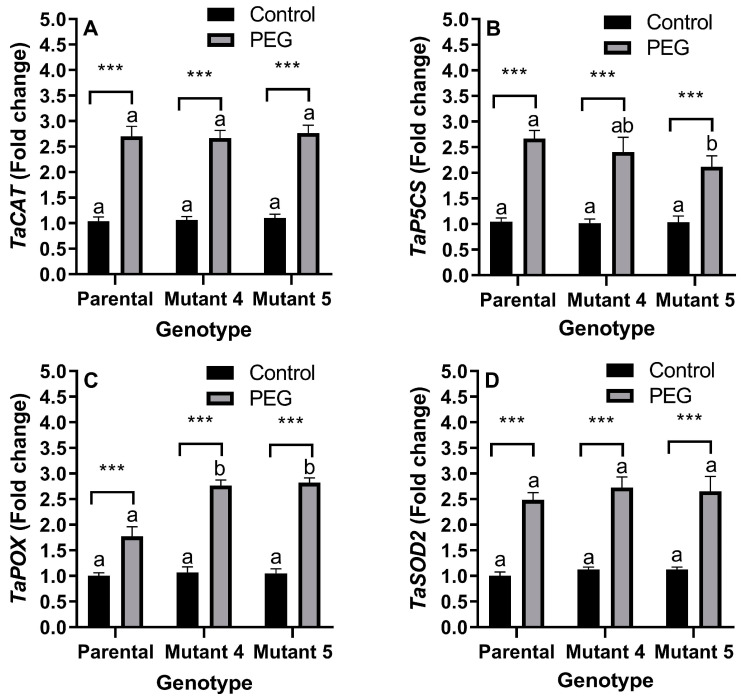
Effects of PEG-induced osmotic stress on the expression of the genes *TaCAT* (**A**), *TaP5CS* (**B**), *TaPOX* (**C**), and *TaSOD2* (**D**) of the parental line (commercial cultivar) and its mutant lines 4 and 5. Values are the arithmetic means of biological triplicates (*n* = 3), and the error bars correspond to the standard deviations (sd). Columns indicated by different letters are statistically different (*p* < 0.05) at the same conditions (control or PEG). Significant differences were determined according to Tukey’s HSD test at *** *p* < 0.001.

PEG treatment significantly (*p* < 0.01) increased while genotype did not affect (*p* > 0.05) the expression of the *TaCAT* gene. There was no interaction (*p* > 0.05) between treatment and genotype for *TaCAT* expression. PEG treatment caused the highest (159.61%) increase in *TaCAT* expression in the parental cultivar (Figure 3A).

PEG treatment significantly (*p* < 0.01) increased and genotype significantly (*p* < 0.05) affected the expression of the *TaP5CS* gene. There was no interaction (*p* > 0.05) between treatment and genotype for *TaP5CS* expression. PEG treatment caused the highest (155.09%) increase in *TaP5CS* expression in the parental cultivar (Figure 3B).

PEG treatment significantly (*p* < 0.01) increased and genotype significantly (*p* < 0.01) affected the expression of the *TaPOX* gene. There was a significant (*p* < 0.01) interaction between treatment and genotype for *TaPOX* expression. PEG treatment caused the highest (168.03%) increase in *TaPOX* expression in the mutant line 5 (Figure 3C).

PEG treatment significantly (*p* < 0.01) increased while genotype did not affect (*p* > 0.05) the expression of the *TaSOD2* gene. No statistically significant (*p* > 0.05) interaction was found between treatment and genotype for *TaSOD2* expression. PEG treatment caused the highest (148.66%) increase in *TaSOD2* expression in the parental cultivar (Figure 3D).

### 2.5. Proteomic Changes

A comparative 2-DE-based proteomic study was performed to evaluate the alterations in protein profiles of three lines under control and drought (PEG) conditions. 2-DE gel maps were produced as shown in Figures S2–S4. A total of 1180 spots were detected when the gels were analyzed by automated spot detection. 62.26% identical protein spots were identified between drought-stressed mutant line 4 and its control, whereas 51.60% were between drought-stressed mutant line 5 and its control (Table 1). The results filtered by the software were as follows: if their probability was ≥95 and 99% and consisted of two or more identified peptides, the peptide and protein identifications were accepted. The false discovery rate (FDR) threshold was set at 0.01 (strict), and the target FDR (relaxed) was 0.05 using the percolator node. Eight spots (A1, B3, B5, B6, B7, C1, C2, C3) had numerous proteins that had score sequence HT (SSHT) values of zero and therefore were not evaluated (Table S3). The remaining 12 spots gave a total of 8 protein matches. Five proteins were detected in the PEG-treated groups of the three lines, while three proteins were detected in the control groups of the parental line and the mutant line 4. Mutant line 4 and the parental line had two and one up-regulated proteins, respectively. All two DEPs were up-regulated in the mutant line 5. Mutant line 4 and the parental line had two and one down-regulated proteins, respectively. Proteins that were detected in the PEG-treated and control groups were listed in Table 2 and Table 3, respectively.

An uncharacterized protein (A0A3B6LWV1) was detected in two spots. One of these spots (C4) was in the control group of the parental line with a SSHT value of 30, and the other spot (B4) was in the PEG-treated group of the mutant line 5 with a SSHT value of 26. This protein was classified as up-regulated in mutant line 5 and down-regulated in the parental line. It has a disordered region; is localized in the “PSI reaction center, GO:0009538”, and involved in “photosynthesis, GO:0015979), with no defined MF. This protein belongs to the *PsaE* family and is highly similar to a PSI reaction center subunit IV protein (A0A9R0XLR3) identified in *T. durum* [26]. Another PSI reaction center subunit IV B protein (Q9S714) has “protein domain specific binding, GO:0019904” function in *A. thaliana* [27]. A0A3B6LWV1 is unstable with an instability index of 43.55 and hydrophilic with a grand average of hydropathicity (GRAVY) index of -0.418 [28,29].

Ribulose bisphosphate carboxylase (Rubisco) large chain (P11383, EC: 4.1.1.39) was detected in four spots with various SSHT values (49, 5, 13, and 34). Three of these spots (A2, A3, and A5) were in the control group of the mutant line 4, and only one spot (A8) was in the PEG-treatment group of the mutant line 4. For this reason, this protein was evaluated as down-regulated in the mutant line 4. Rubisco large chain is encoded by the chloroplast genome and is involved in the “reductive pentose-phosphate cycle, GO:0019253” with its “magnesium ion binding, GO:0000287”, monooxygenase (GO:0004497), and “ribulose-bisphosphate carboxylase, GO:0016984” functions [30]. This protein is unstable with an instability index of 44.03 and hydrophilic with a GRAVY index of −0.278 [28,29].

ATP synthase subunit beta (A0A3B6DE81, EC:7.1.2.2) was detected in the A4 spot of the PEG-treatment group of the mutant line 4. This protein has an AAA+ ATPase domain; is located in the “mitochondrial proton-transporting ATP synthase complex, GO:0005753”; and is involved in “proton motive force-driven mitochondrial ATP synthesis, GO:0042776”, with its “ATP binding, GO:0005524”, and “proton-transporting ATP synthase, GO:0046933” functions [31]. This protein is unstable with an instability index of 43.08 and hydrophilic with a GRAVY index of −0.083 [28,29].

Aminomethyltransferase (A0A3B6CFK1, EC: 2.1.2.10) was detected in the two spots (B1 and B2) of the PEG-treatment group of the mutant line 5 with SSHT values of 19 and 20, respectively. This protein has an aminomethyltransferase folate-binding domain and a glycine cleavage T-protein C-terminal barrel domain; is located in the “mitochondrion, GO:0005739”; is involved in the “glycine catabolic process, GO:0006546” and performs “methyltransferase (GO:0004047)” and “transaminase, GO:0008483” functions [32]. This protein is stable with an instability index of 39.12 and hydrophilic with a GRAVY index of −0.131 [28,29].

Thioredoxin-dependent peroxiredoxin (A0A0C4BJ55, EC:1.11.1.24) was detected in the A7 spot of the PEG-treatment group of the mutant line 4. This protein has a thioredoxin region and a disordered region; it is involved in “response to oxidative stress, GO:0006979” and “cell redox homeostasis, GO:0045454” with its “thioredoxin peroxidase, GO:0008379” function [33]. This protein is stable with an instability index of 36.97 and hydrophilic with a GRAVY index of −0.015 [28,29].

Peroxidase (A0A3B6TLZ2, EC:1.11.1.7) was detected in the C5 spot of the PEG-treatment group of the parental line. This protein has a plant heme peroxidase family profile domain and a disordered region; is located in the “plant-type cell wall, GO:0009505”; is involved in the “hydrogen peroxide catabolic process, GO:0042744”, and “response to oxidative stress” with its “lactoperoxidase, GO:0140825”, “metal ion binding, GO:0046872” and “heme binding, GO:0020037” functions [34]. This protein is stable with an instability index of 38.84 and hydrophobic with a GRAVY index of 0.029 [28,29].

The dihydrolipoamide acetyltransferase component of the pyruvate dehydrogenase complex (A0A3B6LWF0, EC:2.3.1.-) was detected in the A6 spot of the control group of the mutant line 4. This protein has a lipoyl-binding domain, a peripheral subunit-binding domain, and a disordered region; it is involved in the “acetyl-CoA biosynthetic process from pyruvate, GO:0006086”, with its “dihydrolipoyllysine-residue acetyltransferase, GO:0004742” function [35]. This protein is unstable with an instability index of 45.40 and hydrophobic with a GRAVY index of 0.183 [28,29].

**Figure 4:**
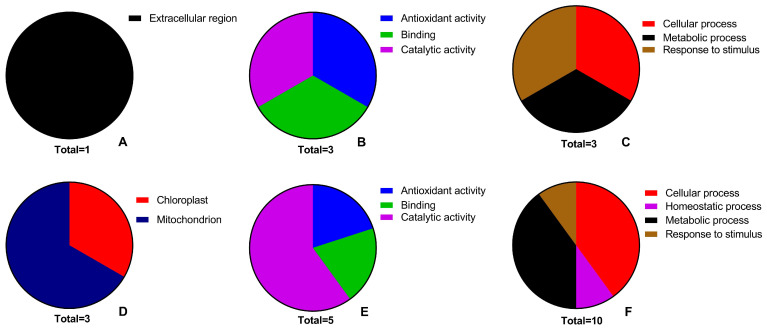
Gene ontology (GO) analyses. Pie charts show the PEG-induced GO terms of cellular component (**A**,**D**), molecular function (**B**,**E**) and biological process (**C**,**F**) in the parental line (**A**–**C**) and in the mutant lines (**D**–**F**).

**Figure 5:**
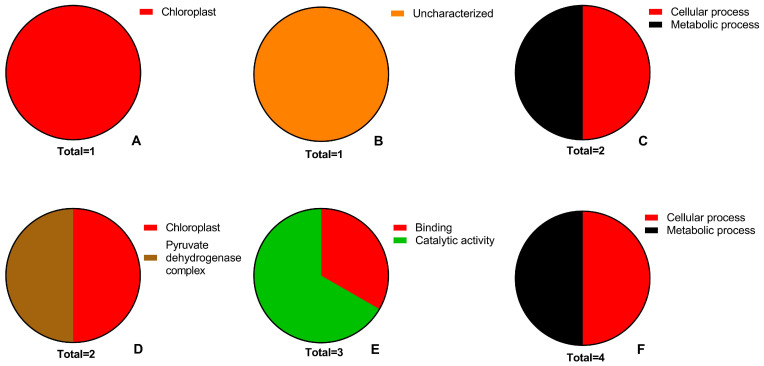
Gene ontology (GO) analyses. Pie charts show the PEG-repressed GO terms of cellular component (**A**,**D**), molecular function (**B**,**E**) and biological process (**C**,**F**) in the parental line (**A**–**C**) and in the mutant lines (**D**–**F**).

Three CC terms were assigned to the up-regulated proteins with one and three matches in the parental line and the mutant lines, respectively (Figure 4A,D). Up-regulated proteins in the mutant lines fell into two categories: “chloroplast, GO:0009507” or “mitochondrion, GO:0005739”. One up-regulated protein in the mutant line 4 has no CC term. One protein that is destined to “extracellular region, GO:0005576), was up-regulated in the parental line. Two CC terms were assigned to the down-regulated proteins with one and two matches in the parental line and the mutant lines, respectively (Figure 5A,D). Down-regulated proteins in the mutant lines fell into two categories: “chloroplast” and “PDC, GO:0045254”. One protein that is destined to “chloroplast” was down-regulated in the parental line.

Three MF terms were assigned to the up-regulated proteins with three and five matches in the parental line and the mutant lines, respectively (Figure 4B,E). Three terms, “antioxidant activity, GO:0016209“, “binding, GO:0005488”, and “catalytic activity, GO:0003824”, were common in all lines. An uncharacterized protein with no defined MF term was up-regulated in the mutant line 5. Two MF terms were assigned to the down-regulated proteins with three matches in the mutant line 4 (Figure 5B). An uncharacterized protein with no defined MF term was down-regulated in the parental line (Figure 5E).

Four BP terms were assigned to the up-regulated proteins with 3 and 10 matches in the parental line and the mutant lines, respectively (Figure 4C,F). Three terms, “cellular process, GO:0009987”, “metabolic process, GO:0008152” and “response to stimulus, GO:0050896” were common in all lines. One protein is also involved in the “homeostatic process, GO:0042592”, in addition to the shared terms. Two BP terms were assigned to the down-regulated proteins with two and four matches in the parental line and the mutant line 4. “cellular process” and “metabolic process” were common to down-regulated proteins in both (the parental line and the mutant line 4) lines, respectively (Figure 5C,F).

### 2.6. Kappa Values

Most of the Kappa values between BP terms that match to the DEPs were either zero or not available. However, “response to stimulus” had a perfect agreement (Kappa score of 1) with “homeostatic process” in the mutant lines (Table S4) due to the thioredoxin-dependent peroxiredoxin protein. When the DEPs of all lines were evaluated, this score was calculated as −0.31 (Table S5).

### 2.7. STI Values

STI values of 3 lines for 10 traits were calculated (Table S6). It is the first time that biochemical measurements, e.g., SOD activity and gene expression data, e.g., *TaP5CS* fold change, were used for the assignment of stress tolerance. *TaCAT* resulted in the highest mean STI of 2.5423. Chlorophyll content gave the lowest mean STI value of 0.7526. The parental line had the highest mean STI value of 2.1236 and had the highest STIs for eight traits, e.g., proline content. The mutant line 5 had the highest STIs for two traits: chlorophyll content and *TaPOX* fold change. The heatmap generated using the STI values indicates that *TaSOD2* and *TaCAT* fold changes are clustered and SOD and CAT enzyme activities formed a distinct cluster. The lines used in this study are classified into two groups, one of which is the parental line, and the mutant lines 4 and 5 form the second group (Figure S5).

## 3. Discussion

In this study, biochemical and proteomic changes induced by in vitro drought stress in seventh generation bread wheat mutants, whose drought tolerance capacity had already been improved by our group [20] using gamma-ray irradiation, were comparatively investigated. For this purpose, drought stress was induced by PEG application under in vitro conditions for 14 days, and then 2 mutant lines and the commercial parental cultivar, from which the mutant lines were derived, were grown in this environment. In order to determine whether drought stress caused any changes in the experimental groups, namely FW and PL, and the contents of chlorophyll, TBARS, and proline, an osmolyte, were measured, and then the activities of antioxidant enzymes such as CAT, POX, and SOD were measured as defense responses of the plants against drought stress. Then, proteomics analyses, i.e., 2-DE and MS, were performed for more detailed analyses of the response to drought stress. Growth-inhibiting effects of the drought were more prominent in the parental line than in the mutant lines, which indeed exhibited a decrease in FW under drought.

### 3.1. Chlorophyll and TBARS Contents

Chlorophyll and the end products of lipid peroxidation contents are indicators of stress-derived cell damage on bio-membrane due to the stress treatments that have been frequently used in recent years [20]. The main effects of drought stress are executed through ROS, which are produced by stress as well as normal cellular processes, e.g., oxidative phosphorylation and photosynthesis [36]. ROS causes damage to biomolecules, e.g., chlorophyll, lipids, proteins, and DNA [8]. High levels of stress decrease chlorophyll content [37,38], and chlorophyll degradation is necessary to avoid cell damage [39]. In this study, PEG treatment decreased chlorophyll content in the parental line and mutant line 4 but not in mutant line 5. Photosynthetic machinery seems to be maintained in the mutant lines under PEG treatment. Drought-tolerant cultivars of bread wheat exhibit less reduction in chlorophyll content, which is used for screening and selection of drought tolerance in bread wheat [40]. An increase in TBARS content as the end product of lipid peroxidation is another phenomenon under stress treatments [41]. In this study, mutant lines showed less increase in TBARS under drought than in the parental line. This situation showed that the defense systems in the mutant lines cope with lipid peroxidation in cell membranes compared to the parental genotype. Relatively less degradation of chlorophyll in the mutant lines compared to the parental genotype supports this finding.

### 3.2. Antioxidant Enzyme Activities and Proline Content

Increasing antioxidant enzyme activities and proline content are bio-indicators for stress response [20,42]. Under physiological conditions, plants modulate ROS levels through enzymatic, e.g., CAT, POX, and SOD, and non-enzymatic, e.g., proline, compounds. Proline, an antioxidant, an osmolyte, and also an energy provider, accumulates in plants under drought stress. It has been demonstrated that an exogenous proline supplement can enhance drought tolerance in maize [9]. SOD is the first line of defense against ROS and the most effective enzymatic antioxidant. It decomposes superoxide radicals and releases H_2_O_2_ and O_2_ [18]. In several species, such as *Oryza sativa*, SOD is usually activated by drought stress. H_2_O_2_ is eliminated by CAT and by POX [8]. In the present study, PEG treatment stimulated the antioxidant system in all lines, and POX was the most activated enzyme. The mutant lines 4 and 5 had the highest proline level and CAT activity even under control conditions, respectively. These results can be inferred as the enhancement of CAT, POX, and SOD enzymes was insufficient to prevent the harmful effects of ROS, particularly in the parental line, but the mutant lines might have been protected from stress treatments by other components of the vast antioxidant system. As with the membrane damage bioindicators mentioned above, having an advanced defense system is also a frequently used criterion in selection against stress in plants [20].

### 3.3. Transcriptional Regulation

The changes in enzyme activity do not always correlate with levels of the corresponding mRNA transcripts [43]. For this reason, we measured the expression levels of the *TaCAT*, *TaPOX*, *TaP5CS*, and *TaSOD2* genes. Drought induces the transcription of the genes encoding CAT, P5CS, and SOD in bread wheat, yet the increments are more prominent in the drought-tolerant variety [44]. Activation of the gene encoding P5CS was suggested to be a marker of drought stress in wheat [45]. The genes encoding POX are also induced upon salinity and drought and confer stress tolerance in wheat [45,46]. As expected, in terms of fold change levels, *TaCAT* was the most induced gene, while *TaPOX* had fold change values over two in the mutant lines, making it an indicative gene. However, the highest induction of the *TaPOX* gene in the mutant lines does not correlate with the activity of its enzyme, which reached its highest activity in the parental line under PEG treatment. Induction levels of the *TaSOD2* gene were similar in all genotypes, while its enzyme was most activated in the parental line. The *TaP5CS* gene was more up-regulated by PEG treatment in the parental line than in the mutant lines. This is consistent with the highest proline accumulation in the PEG-treated parental line. The changes in gene expression except for *TaP5CS* were more significant than the changes in their biochemical activities. This can be attributed to the PEG-induced disruptions in protein translation and post-translational mechanisms. Similar observations were made in both plant [47] and mammalian cells under stress [43].

### 3.4. Chloroplast Proteins

GO is a structured ontology that classifies genes into three main categories: BP, MF, and CC. BP describes molecular event processes, MF relates to gene functions at the molecular level, and CC refers to cellular locations or environments [48]. In this study, DEPs in both parental and mutant lines are predominantly associated with chloroplasts or mitochondria.

The photosynthetic apparatus includes PSI and PSII. PSI, with about 175 chlorophyll molecules and at least 19 protein subunits, including PsaE, transfers electrons from plastocyanin to ferredoxin, generating a strong redox potential [49]. Ferredoxin-NADP+-oxidoreductase then converts NADP+ to NADPH, which, along with ATP, is used for CO_2_ fixation [50]. CO_2_ deprivation and high irradiance cause downregulation of photosynthesis, overreduction of reaction centers, and ROS formation, leading to photooxidation. In Cyanobacteria, reduced plastoquinone (PQ) activates PSI gene transcription, while oxidized PQ activates PSII genes [51]. Reduced PQ also prevents lipid peroxidation and protects PSII [52]. In drought-tolerant wheat, PSI subunits were up-regulated by osmotic stress [53]. Luo et al. [54] linked PSI subunit up-regulation to maintained PSI activity under heat stress. A PSI-related protein from the PsaE family was up-regulated in mutant line 5 but down-regulated in the parental line, indicating improved photosynthetic capacity in the mutant line under osmotic stress.

Rubisco, a key enzyme for carbon fixation, transfers CO_2_ and H_2_O to ribulose 1,5-bisphosphate to produce 3-carbon phosphoglyceric acid [55]. Photosynthesis-related genes impact stress responses [38,56], with drought reducing Rubisco abundance in both sensitive and tolerant rice cultivars. However, tolerant cultivars have mechanisms to maintain Rubisco activity [57]. In this study, Rubisco abundance in mutant line 4 under control conditions suggests efficient carbon fixation. The presence of Rubisco in one spot under PEG conditions may reflect drought-induced protein modifications. Variations in protein spots may result from post-translational modifications and proteolytic cleavage [58,59].

### 3.5. Mitochondrial Proteins

Expression of ATP synthesis-related proteins is enhanced in plants under osmotic stress [60]. Mitochondrial ATP synthase, comprising hydrophilic F0 and F1 components with three beta subunits, utilizes proton gradients to phosphorylate ADP to ATP and can also hydrolyze ATP and pump protons through the inner mitochondrial membrane [61]. Plant mitochondria are crucial in stress responses due to their role in energy production and ROS generation. Drought-induced inhibition of photosynthesis is offset by increased mitochondrial ATP production, essential for cellular homeostasis [62]. Enrichment of mitochondrial ATP synthase may enhance drought tolerance [63]. In Tibetan wild barley, the beta subunit of chloroplastic ATP synthase was up-regulated in drought-tolerant genotypes but down-regulated in sensitive ones [64]. Similarly, ATP synthase CF1 alpha subunit was repressed by drought in bread wheat [58]. Enhanced ATP synthase activity likely contributes to the relative drought tolerance observed in the mutant line 4.

The glycine cleavage system (GCS), consisting of four proteins, including aminomethyltransferase (T-protein), degrades glycine to NH_4_^+^, CO_2_, and a methylene group, consuming NAD^+^ and producing NADH. 2-phosphoglycolate, produced by Rubisco’s oxygenase activity, is converted to glycine in peroxisomes and then transported to mitochondria. There, it is processed by GCS and serine hydroxymethyl transferase to produce serine, which is converted to 3-phosphoglycerate for the Calvin cycle [65,66]. Drought-induced up-regulation of aminomethyltransferase was observed in drought-tolerant *Pennisetum glaucum* and *Euterpe oleracea* [67,68], suggesting that glycine catalysis helps maintain photosynthesis and NADH production in mutant line 5.

### 3.6. Other Proteins

High photon intensity can increase ROS production, which can damage chloroplasts if not eliminated [69]. Peroxiredoxins, along with other enzymes such as CAT and APX, are crucial for detoxifying ROS and protecting cellular components, especially in chloroplasts [70,71]. They also maintain genomic stability and are essential for stress tolerance [72,73]. The accumulation of peroxiredoxins under osmotic stress in mutant line 4 might enhance its antioxidant defense, consistent with lower TBARS levels observed in this line.

Class III POXs, which are abundant in cell walls and involved in cell wall modification, increase in response to stress [74,75]. Drought stress can upregulate POXs in stress-tolerant wheat varieties, though findings can vary [76]. In our study, POX was detected in the parental line under PEG stress but not in drought-tolerant mutants, indicating differences in stress response [77].

Pyruvate dehydrogenase complex (PDC) converts pyruvate to acetyl-CoA, influences energy production and stress responses. PDC components, including dihydrolipoamide transacetylase (E2), are crucial for its activity [78,79]. The downregulation of E2 in mutant line 4 under PEG treatment suggests efficient PDC activity contributing to stress tolerance [80,81].

Intrinsically disordered proteins (IDPs) have flexible structures that facilitate various cellular functions. IDPs, including Late-Embryogenesis Abundant proteins and dehydrins are involved in stress responses [82,83]. In our study, several DEPs with disordered regions were identified. Two (A0A3B6LWV1, A0A0C4BJ55) and one (A0A3B6TLZ2) of them were up-regulated in mutant lines and the parental line, respectively. A0A3B6LWV1 and A0A3B6TLZ2 are unstable proteins with instability indices higher than 40. Differential expression of IDPs might reflect their role in stress adaptation [84].

### 3.7. Relationship between BP Terms

Cohen’s Kappa statistic measures the agreement between two observers [85]. In biological and clinical studies, examining the associations between variables is crucial [86]. In GO analyses, the Kappa score assesses the interaction between two terms, grouping them into functional categories based on shared genes [87,88]. Terms with numerous common genes can form a module or a relationship network [89]. For the DEPs in mutant lines 4 and 5, “response to stimulus” showed perfect agreement with “homeostatic process”. However, no related BP terms were identified for DEPs across all three lines. This suggests that “response to stimulus” and “homeostatic process” share similar gene regulation in the mutant lines, potentially contributing to their drought tolerance.

### 3.8. STI Clustering

Several indices can identify stress-tolerant genotypes, with the Stress Tolerance Index (STI) being one of them [90]. STI can be calculated for various traits such as root/shoot/coleoptile length, dry matter, germination rate, grain yield, and chlorophyll fluorescence [91,92,93,94]. Genetic fingerprinting data indicate that mutant lines 4 and 5 are more closely related to each other than to the parental line [20]. In this study, STI values showed that these mutant lines are genetically closer. Drought-tolerant genotypes generally have higher STIs, and antioxidant enzyme activities such as APX and CAT can help assess stress tolerance [92]. Previous reports demonstrated that these mutant lines performed better than the parental line, especially in antioxidant capacity [20]. However, the STI formula in this study only predicted drought tolerance for chlorophyll content and *TaPOX* expression traits. In contrast, our previous paper [20] assessed biochemical performance in a bulk of 11 mutant lines, including the two studied here, which could explain the differences in STI values.

## 4. Materials and Methods

### 4.1. Plant Material

The parental line used in this study is the bread wheat (*T. aestivum* L.) cultivar Sagittario and was obtained from the Trakya Agriculture Research Institute in Edirne, Turkey. Sagittario is a wheat variety that is moderately tolerant to drought [95]. Gamma irradiation of the parental line and obtaining its M_4_ generation mutant lines were already explained Sen et al. 2017 [20]. Briefly, seeds were exposed to 6.5 Gy per min at a dose of 200 Gy. M_1_ and M_2_ generations were grown at the Trakya Agriculture Research Institute under field conditions. Immature M_3_ generation seeds were grown in vitro to generate the M4 generation. Selection was performed until the seventh generation. Immature seeds of the sixth generation (M6) were collected, and their embryos were isolated under aseptic conditions. The embryos were transferred to 0.5× MS medium supplemented with 20 gL^−1^ sucrose, 30 gL^−1^ PEG 6000, and 8 gL^−1^ agar and incubated in a growth chamber for a 16 h light/8 h dark photoperiod with an irradiance of 500 μmol m^−2^ s^−1^ photon flux density at 26 °C for 14 days. Control plants were transferred to the same MS medium without PEG. At the end of two weeks, the weights of the regenerated plantlets were individually weighed and the lengths of the regenerated plantlets were individually measured to calculate the average plant fresh weight and the average plantlet length in the experimental groups, respectively. Leaf samples of the plantlets were flash frozen in liquid nitrogen and kept at −70 °C until further use.

### 4.2. Measurement of Total Chlorophyll, Proline and the End Products of Lipid Peroxidation Contents

Leaf tissues were used for the biochemical experiments. The method of Arnon [96] was used to measure total chlorophyll content by spectrophotometry. The ninhydrin method was used for the determination of proline content [97]. The end products of lipid peroxidation were measured as the amount of thiobarbituric acid-reactive substances (TBARS) determined by the thiobarbituric acid (TBA) reaction as described by Heath and Packer [98].

### 4.3. Measurement of the Enzyme Activities

200 mg of frozen leaf tissue was extracted using an extraction buffer containing 100 mM phosphate buffer (pH 7.0), 1% polyvinylpyrrolidone 40 (PVP40) (*w*/*v*), and 0.1 mM disodium ethylenediaminetetraacetate dihydrate (Na_2_-EDTA) to measure the activities of antioxidant enzymes. Homogenates were centrifuged at 13.000× *g* for 25 min at 4 °C, and the supernatants were used for further analysis. Protein content was measured according to Bradford [99]. Superoxide dismutase (SOD, EC:1.15.1.1) activity was assayed by monitoring the superoxide radical-induced nitro blue tetrazolium chloride (NBT) reduction at 560 nm [100]. One unit of SOD activity was characterized as the amount of enzyme, which leads to 50% inhibition of the photochemical reduction of NBT. The measurement of peroxidase (POX, EC:1.11.1.7) activity was measured at 470 nm by using (H_2_O_2_), and guaiacol as substrates. The disappearance of H_2_O_2_ was monitored at 240 nm for the determination of catalase (CAT, EC:1.11.1.6) activity [101].

### 4.4. qRT-PCR Analysis

RNA was isolated from the frozen leaves by TRIzol reagent. The specific primers (Table S7) were designed using Primer 3, a web-based tool [102]. mRNA sequences from target genes were retrieved from GenBank (National Center for Biotechnology Information) and loaded on Primer3 to design the primers using the default parameters, as melting temperatures ranged between 60 and 65 °C and the primer and DNA amplicon lengths were ranged between 18 and 24 bp and 100 and 300 bp, respectively. Subsequently, the cDNA was synthesized via the Superscript II reverse according to the manufacturer’s instructions. The qRT-PCR was performed on a Light Cycler Nano apparatus using SYBR Green Master Mix. Actin (*TaActin*) genes from *T. aestivum* were used as internal controls. The relative gene expression was calculated using the 2^−ΔΔCt^ formula [103].

### 4.5. Protein Extraction

Total proteins were extracted from leaves using a TCA/acetone method [104]. 0.3 g of leaf tissues were ground by liquid nitrogen into fine powder using a pestle and a mortar. Samples were extracted with 10% TCA and 1% DTT containing acetone. After storage at −20 °C for 1 h, the samples were centrifuged at 25,000× *g* for 20 min. at 4 °C. The pellet was washed in acetone containing 1% DTT and incubated at −20 °C for 1 h to rinse the pigments from the samples. This step was repeated until a colorless sample pellet was obtained. The colorless pellet was vacuum dried and dissolved in 8 M urea buffer containing 20 mM DTT, 4% CHAPS, and 2% ampholyte (pH 3–10) by vortexing for 2 min at RT. The solution was centrifuged two times at 25,000× *g* at room temperature for 5 min. The supernatant that contains the protein mixture was collected, and its protein content was quantified by the Bradford method [99].

### 4.6. 2DE Analysis

Isoelectric focusing was performed on the Protean IEF system using 17 cm ReadyStrip™ IPG strips. Protein samples were prepared by mixing rehydration/sample buffer to achieve a protein concentration of 405 ng in 300 µL. Rehydration was carried out at 20 °C with a constant current of 50 V for 12 h, followed by 100 V for 1 h, 200 V for 1 h, 400 V for 16.5 h, and 500 V for 2.5 h. After the first dimension focusing, separation of proteins by molecular weight was performed following equilibration of the IPG strips with equilibrium buffer (1/2). The equilibrium buffer contained 6 M urea, 2% SDS, 0.375 M Tris-HCL (pH 8.8), 20% glycerol, 2% DTT, and 2.5% iodoacetamide. The separation was performed on 22 × 24 cm 12% precast SDS-PAGE gels (distilled water, acrylamide/bisacrylamide (30%, 0.8% *w*/*v*), 12% SDS, 1.5 M Tris-HCL pH 8.8, TEMED, 10% APS. Samples were run at a constant current of 30 mA for 30 min and then at 4 mA for 6 h. After the second-dimension separation, the samples were fixed in a solution of 50% ethanol and 2% phosphoric acid for 2 h. The gels were then transferred to a staining solution containing 34% methanol, 2% phosphoric acid, 17% ammonium sulfate, and 0.05% Coomassie blue. The gels were visualized and analyzed using the PDQuest Advanced gel analysis software (1709620). The level of protein expression was determined by comparing each dose with the control and assessing the increase or decrease in protein spots. The spots identified were cut from a preparative gel with an automated spot-cutting instrument and subjected to digestion by an in-gel tryptic cleavage kit followed by MALDI-TOF/TOF analysis.

### 4.7. LC-MS/MS Data Analysis

The peptide sample obtained after cleavage was loaded onto the Ultimate 3000 RSLC nano ultra HPLC system. The peptide samples were chromatographically separated on the Ultimate 3000 RSLC nano ultra HPLC system (ULTIM3000RSLCNAN) and analyzed on the Q Exactive mass spectrophotometer (IQLAAEGAAPFALGMBDK). A C18 capture column was used to remove the concentration of peptides and impurities such as salts, and then the peptide samples were transferred to an Acclaim PepMap RSLC C18 analytical column (75 μm × 15 cm × 2 μm, 100 Å diameter) for chromatographic separation using mobile phase A (0.1% formic acid) and mobile phase B (80% acetonitrile), 0.1% formic acid. MS and MS/MS analyses of the samples were performed on a Q Exactive mass spectrophotometer for 45 min. The raw data sets obtained from the analyses were analyzed in Proteome Discoverer 2.2. First, an up-to-date protein database was created using Uniprot/Swissprot. Using this database, the peptides were analyzed according to established parameters (peptide mass tolerance 10 ppm, MS/MS mass tolerance 0.2 Da, mass accuracy 2 ppm, minimum peptide length 6 amino acids, stable modifications such as cysteine carbamidomethylation, and unstable modifications such as methionine oxidation and asparagine deamination). To identify the proteins, the setting parameters were chosen as in Albayrak et al. [105]. The search results filtered by the software were as follows: if their probability was ≥95 and 99% and consisted of two or more identified peptides, the peptide and protein identifications were accepted. The false discovery rate (FDR) threshold was set at 0.01 (strict), and the target FDR (relaxed) was 0.05 using the percolator node. Information about the proteins that were taken into consideration was retrieved from the UniProt Knowledgebase [106] and ProtParam [29] databases.

### 4.8. Statistical Analyses

The experiments to measure chlorophyll, proline, and TBARS content and the activities of CAT, POX, and SOD enzymes were conducted five times, and, therefore, each datapoint is the arithmetic mean of biological pentaplicates (*n* = 5). Gene expression analyses were performed three times, and, therefore, each datapoint is the arithmetic mean of biological triplicates (*n* = 3). Data were analyzed by 2-Way ANOVA and the Tukey’s multiple comparison test using Graphpad Prism (version 8.0.1.244). The ANOVA analyses included 2 independent variables as genotype (parental, mutant 4, and mutant 5) and treatment (control and PEG) and 10 dependent variables.

Annotations of the DEPs were performed according to GO Browser [107]. Proteins were grouped into their cellular component (GO:0005575, CC), molecular function (GO:0003674, MF), and biological process (GO:0008150, BP). The immediate child terms of MF and BP were taken into consideration. CC terms, however, were custom generated.

The degree of agreements between BP terms according to the genes regulated in the mutant lines or the 3 lines including the parental line were analyzed by Cohen’s Kappa statistics [107]. Two BP terms with a kappa value bigger than 0.21, 0.41, and 0.61 were considered to have a fair, moderate, and substantial agreement, respectively [108]. A high Kappa score indicates that 2 terms share many common proteins [109].

The stress tolerances of the 3 lines were calculated using the formula “STIi = V_in_/V_ic_” and given as STI. STIi is the tolerance index of any trait, and V_in_ and V_ic_ correspond to the values of that trait in the stressed treatment and control, respectively [93]. STI was calculated for all lines and for the biochemical and molecular traits (10 traits), e.g., proline content and fold change of *TaSOD2*. STI values were further used for the generation of the hierarchical clustering heatmap using R software 4.3.3 [92,110].

## 5. Conclusions

PEG-induced drought stress led to a stress response in all three genotypes, as indicated by the accumulation of proline, and resulted in oxidative stress, despite increased activities of the three main antioxidant enzymes. Increased enzyme activity was correlated with up-regulated transcription of the genes encoding antioxidant enzymes. The parental line and the mutant line 5 showed up-regulated DEPs, while the mutant line 4 also exhibited down-regulated DEPs. Proteins that are located in the chloroplast or mitochondrium indicate the main differences between the parental line and the mutant lines. Antioxidant enzymes are also differentially regulated at the translational level. Drought tolerance of the mutant lines might be attributed to improved photosynthesis and efficient ATP synthesis. Accumulation of thioredoxin-dependent peroxiredoxin in the mutant line 4 might have contributed to its tolerance. “Response to stimulus” and “Homeostatic process” might be key processes involved in drought tolerance in wheat. Chlorophyll content and *TaPOX* gene expression might be indicators of drought tolerance in bread wheat. In light of the data we obtained, our next plan is to continue analyses at the gene level. These findings are expected to be utilized in plant breeding studies.

## Figures and Tables

**Table 1:** Number of spots detected in 2-DE analysis in the parental and mutant plants subjected to drought stress.

Group	Number of Spots in Control Group	Number of Spots in PEG-Treated Group	Number of Shared Spots
Parental line	193	105	62
Mutant line 4	177	106	66
Mutant line 5	318	281	145
Total	688	492	273

**Table 2:** Proteins found in the PEG treatment groups, spot names, IDs, names, amino acid numbers, MWs, pI values, and the line detected.

Spot	ID	Name	Amino Acids	Molecular Weight (kDa)	pI	Detected in
A4	A0A3B6DE81	ATP synthase subunit beta	498	53.9	5.16	Mutant line 4
A7	A0A0C4BJ55	Thioredoxin-dependent peroxiredoxin	258	27.9	6.79	Mutant line 4
A8	P11383	Ribulose bisphosphate carboxylase large chain	477	52.8	6.68	Mutant line 4
B1	A0A3B6CFK1	Aminomethyltransferase	415	44.4	8.57	Mutant line 5
B2	A0A3B6CFK1	Aminomethyltransferase	415	44.4	8.57	Mutant line 5
B4	A0A3B6LWV1	Uncharacterized protein	148	15.6	9.70	Mutant line 5
C5	A0A3B6TLZ2	Peroxidase	358	37.7	6.77	Parental line

**Table 3:** Proteins found in the control groups, spot names, IDs, names, amino acid numbers, MWs, pI values, and the line detected.

Spot	ID	Name	Amino Acids	Molecular Weight (kDa)	pI	Detected in
A2	P11383	Ribulose bisphosphate carboxylase large chain	477	52.8	6.68	Mutant line 4
A3	P11383	Ribulose bisphosphate carboxylase large chain	477	52.8	6.68	Mutant line 4
A5	P11383	Ribulose bisphosphate carboxylase large chain	477	52.8	6.68	Mutant line 4
A6	A0A3B6LWF0	Dihydrolipoamide acetyltransferase component of PDC	475	48.8	8.91	Mutant line 4
C4	A0A3B6LWV1	Uncharacterized protein	148	15.6	9.70	Parental line

## Data Availability

Data is contained within the article or the Supplementary Material.

## Supplementary Materials

The following supporting information can be downloaded at: https://www.mdpi.com/article/10.3390/plants13192702/s1. Figure S1: Fourteen-day-old bread wheat plants of the parental line (A), mutant line 4 (B), and mutant line 5 (C) under control (a) and PEG (b) conditions; Figure S2: 2D spots in the mutant line 4 under control (A) and PEG conditions (B); Figure S3: 2D spots in the mutant line 5 under control (A) and PEG conditions (B); Figure S4: 2D spots in the parental line under control (A) and PEG conditions (B); Figure S5: Hierarchical clustering heatmap graph of the traits and the lines based on the STIs; Table S1. Fresh weight (mg) of the plants under control or PEG conditions. Values are the arithmetic means of biological triplicates (*n* = 3) and the standard deviations (sd) and analyzed by 2-way ANOVA and Tukey’s HSD test. Columns indicated by the same letter “a” are statistically insignificant (*p* > 0.05) at the same conditions (control or PEG). Significant differences of a genotype at different conditions were marked with * *p* < 0.05 or *** *p* < 0.001; Table S2. Plantlet length (cm) of the plants under control or PEG conditions. Values are the arithmetic means of biological triplicates (*n* = 3) and the standard deviations (sd) and analyzed by 2-way ANOVA and Tukey’s HSD test. Columns indicated by the same letter “a” are statistically insignificant (*p* > 0.05) at the same conditions (control or PEG). Non-significant differences of a genotype at different conditions were marked with ns *p* > 0.05; Table S3: Raw 2D data including protein IDs, amino acid numbers, peptides, pI, coverage %, and Score Sequest HT values; Table S4. Agreement scores (Kappa values) between BP terms in the mutant lines; Table S5. Agreement scores (Kappa values) between BP terms in all lines; Table S6. STIs of each line for several traits; Table S7. Sequence of the primers and the accession number of the corresponding genes and their amplicon size (bp).
