# Supplementary material for: Biochemical and Proteomic Analyses in Drought-Tolerant Wheat Mutants Obtained by Gamma Irradiation
Source: Plants (Basel). 2024 Sep 27;13(19):2702. doi: 10.3390/plants13192702 (PMC11478800; doi:10.3390/plants13192702)
Supplement: Supplementary file 1 [file plants-13-02702-s001.zip › plants-3149331-supplementary.pdf]

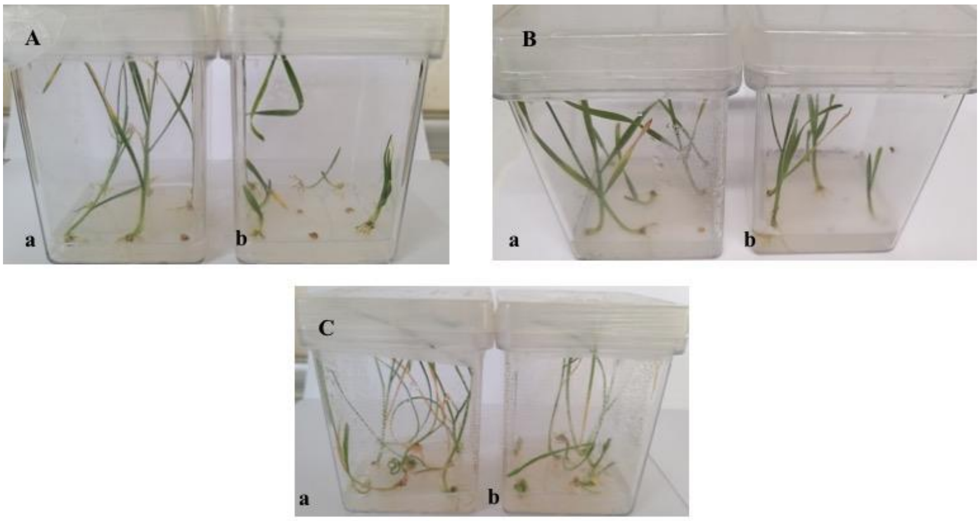

Figure S1. Fourteen-day-old bread wheat plants of the parental line (A), mutant line 4 (B) and mutant line 5 (C) under control (a) and PEG (b) conditions

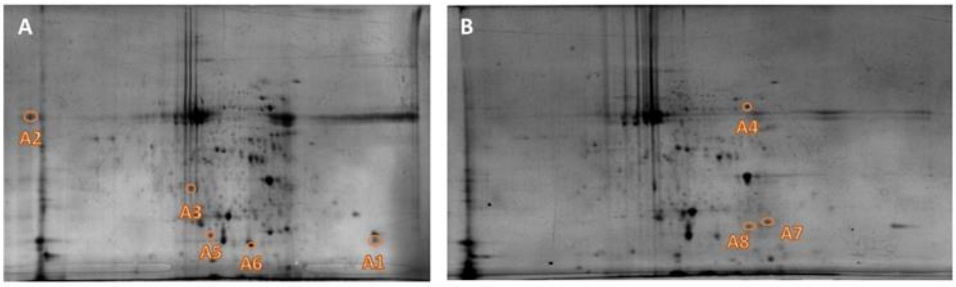

Figure S2. 2DE spots in the mutant line 4 under control (A) and PEG conditions (B)

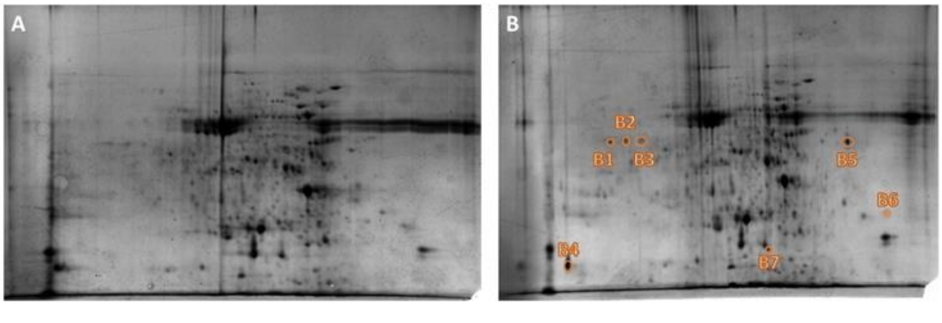

Figure S3. 2DE spots in the mutant line 5 under control (A) and PEG conditions (B)

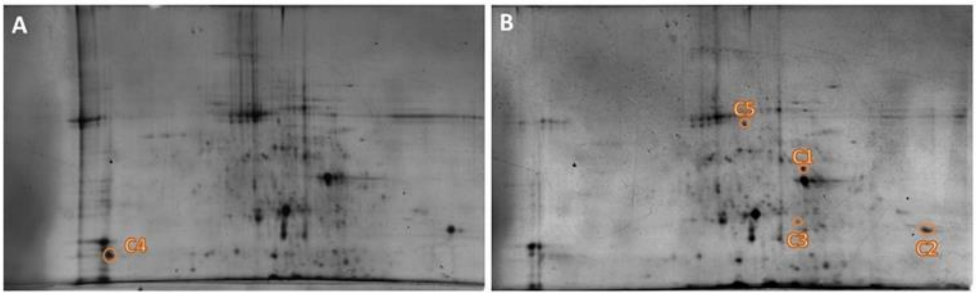

Figure S4. 2DE spots in the parental line under control (A) and PEG conditions (B)

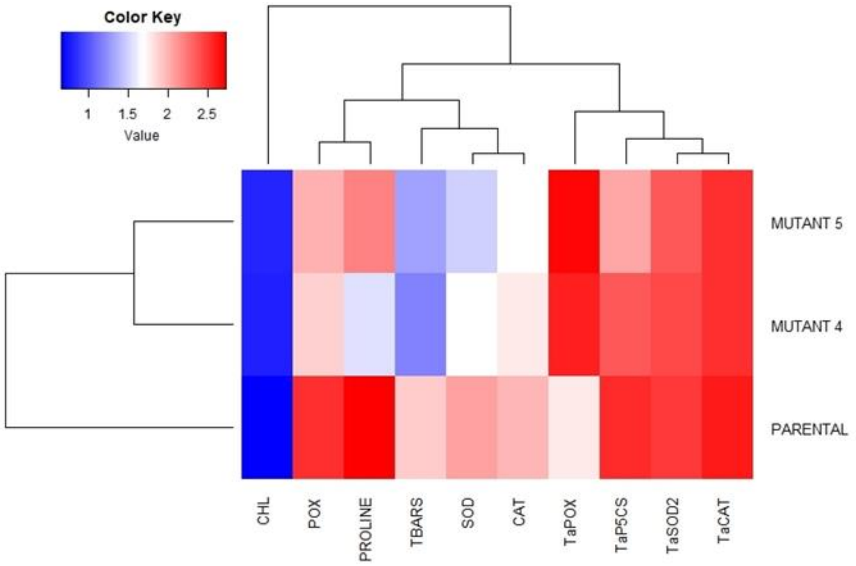

Figure S5. Hierarchical clustering heatmap graph of the traits and the lines based on the STIs

Table S1. Fresh weight (mg) of the plants under control or PEG conditions. Values are the arithmetic means of biological triplicates (total n=30) and the standard deviations (sd) and analyzed by 2-way ANOVA and Tukey's HSD test. Columns indicated by the same letter "a" are statistically insignificant (p > 0.05) at the same conditions (control or PEG). Significant differences of a genotype at different conditions were marked with * p < 0.05 or *** p < 0.001.

	Genotype					
	Parental		Mutant 4		Mutant 5	
	Control	PEG	Control	PEG	Control	PEG
**Mean ± sd**	587 a ± 97	193 a ± 47 ***	575 a ± 103	346 a ± 75 *	613 a ± 73	378 a ± 53 *
**Treatment**	P < 0.001					
**Genotype**	P > 0.05					
**Interaction**	P > 0.05					

Table S2. Plantlet length (cm) of the plants under control or PEG conditions. Values are the arithmetic means of biological triplicates total n=30) and the standard deviations (sd) and analyzed by 2-way ANOVA and Tukey's HSD test. Columns indicated by the same letter "a" are statistically insignificant (p > 0.05) at the same conditions (control or PEG). Non-significant differences of a genotype at different conditions were marked with ns p > 0.05.

	Genotype					
	Parental		Mutant 4		Mutant 5	
	Control	PEG	Control	PEG	Control	PEG
**Mean ± sd**	11.5 a ± 3.5	5.7 a ± 1.4 ns	11.5 a ± 4.2	7.5 a ± 2.2 ns	11 a ± 3.5	7.7 a ± 4.1 ns
**Treatment**	P > 0.05					
**Genotype**	P > 0.05					
**Interaction**	P < 0.05					

Table S3. Raw 2DE data including protein IDs, amino acid numbers, peptides, pI, coverage % and Score Sequest HT values.

Spot	ID	Name	Coverage %	Peptides	PSMs	Unique Peptides	Protein Groups	AAs	MW (kDa)	pI	Score Sequest HT
A1	**A0A3B6NM43**	**Zinc finger GRF-type domain-containing protein**	**67.114**	**1**	**1**	**1**	**1**	**149**	**16.7**	**9.07**	**0.00**
A1	**A0A3B6Q8N2**	**Neprosin domain-containing protein**	**16.085**	**1**	**6**	**1**	**1**	**373**	**41.9**	**8.25**	**0.00**
A1	**A0A3B6DC22**	**3'-5' exonuclease domain-containing protein**	**12.173**	**1**	**2**	**1**	**1**	**575**	**62.2**	**4.91**	**0.00**
A1	**A0A3B6TRG1**	**F-box domain-containing protein**	**6.179**	**1**	**7**	**1**	**1**	**356**	**40.2**	**8.46**	**0.00**
A1	**A0A3B5ZTU6**	**Pentacotripeptide-repeat region of PRORP domain-containing protein**	**0.975**	**1**	**2**	**1**	**1**	**820**	**90.2**	**8.13**	**0.00**
A1	**A0A3B6LGT9**	**CBS domain-containing protein**	**10.731**	**1**	**1**	**1**	**1**	**205**	**23.1**	**8.62**	**0.00**
A2	**A0A3B6LWF0**	**Dihydrolipoamide acetyltransferase component of PDC**	**18.947**	**1**	**3**	**1**	**1**	**475**	**48.8**	**8.91**	**1.77**
A2	**P11383**	**Ribulose bisphosphate carboxylase large chain**	**23.060**	**12**	**64**	**12**	**1**	**477**	**52.8**	**6.68**	**49.03**
A2	**A0A3B6RNL5**	**DNA (cytosine-5)-methyltransferase**	**0.850**	**1**	**1**	**1**	**1**	**1528**	**171.3**	**6.44**	**0.00**

A2	**A0A3B6KQK1**	**Dihydrolipoamide acetyltransferase component of PDC**	**189.873**	**1**	**3**	**1**	**1**	**474**	**48.6**	**9.03**	**1.77**
A2	**A0A3B6N0L8**	**Dihydrolipoamide acetyltransferase component of PDC**	**189.873**	**1**	**3**	**1**	**1**	**474**	**48.6**	**8.75**	**1.77**
A3	**A0A3B5ZPP9**	**S1 motif domain-containing protein**	**0.632**	**1**	**1**	**1**	**1**	**1898**	**210.4**	**8.48**	**0.00**
A3	P11383	Ribulose bisphosphate carboxylase large chain	46.121	3	10	3	1	477	52.8	6.68	5.80
A3	A0A3B6NVZ4	DUF4220 domain-containing protein	50.328	1	2	1	1	457	52.0	9.98	0.00
A3	A0A3B6LWF0	Dihydrolipoamide acetyltransferase component of PDC	18.947	1	3	1	1	475	48.8	8.91	1.72
A3	A0A3B6PEF1	AAA+ ATPase domain-containing protein	37.990	1	2	1	1	816	91.4	8.47	0.00
A3	A0A3B6LLP8	Subtilisin-like protease	45.283	1	1	1	1	795	84.6	8.54	0.00
A3	A0A3B6HZ89	X8 domain-containing protein	74.786	1	1	1	1	468	47.8	5.10	0.00
A3	A0A3B6RQV9	HMA domain-containing protein	27.972	1	1	1	1	1001	106.8	5.47	0.00
A4	A0A3B6TM15	Protein kinase domain-containing protein	28.492	1	2	1	1	1088	116.6	5.55	0.00
A4	A0A3B6DE81	ATP synthase subunit beta	28.514	10	46	10	1	498	53.9	5.16	43.53
A4	Q9SBB7	Chloroplast small heat shock protein	11.934	1	4	1	1	243	26.9	8.12	0.00
A5	A0A3B6NJ19	F-box domain-containing protein	71.038	1	1	1	1	366	41.5	7.90	0.00
A5	P11383	Ribulose bisphosphate carboxylase large chain	56.603	3	16	3	1	477	52.8	6.68	13.93
A6	A0A3B6IWY8	Agglutinin domain-containing protein	19.120	1	2	1	1	523	59.0	5.68	0.00
A6	A0A3B6LWF0	Dihydrolipoamide acetyltransferase component of PDC	18.947	1	2	1	1	475	48.8	8.91	1.82
A6	A0A3B5XYG4	Secretory protein	1.148	1	1	1	1	235	25.3	4.89	0.00
A6	A0A3B6LUR4	Bifunctional inhibitor/plant lipid transfer protein/seed storage helical domain-containing protein	40.609	1	5	1	1	197	19.6	8.13	0.00
A6	A0A3B6CFY2	Amine oxidase domain-containing protein	0.918	1	1	1	1	871	97.5	7.75	0.00
A6	A0A3B6SQ24	Chalcone synthase	26.755	1	2	1	1	299	32.4	6.61	0.00
A7	A0A3B6PEF5	PGG domain-containing protein	1.048	1	2	1	1	572	63.0	8.84	0.00
A7	A0A0C4BJ55	thioredoxin-dependent peroxiredoxin	20.155	7	73	1	1	258	27.9	6.79	43.88
A7	A0A3B6GUH6	Uncharacterized protein	10.276	1	5	1	1	1265	137.5	7.77	0.00
A7	A0A3B5XTS3	tRNA pseudouridine(55) synthase	1.600	1	1	1	1	500	56.1	8.94	0.00
A7	A0A3B6LUR4	Bifunctional inhibitor/plant lipid transfer protein/seed storage helical domain-containing protein	40.609	1	4	1	1	197	19.6	8.13	0.00
A7	A0A077RXY2	ABC transporter domain-containing protein	26.446	1	2	1	1	605	65.3	9.57	0.00

A7	A0A3B6C702	thioredoxin-dependent peroxiredoxin	19.847	7	70	1	1	262	28.3	6.79	37.71
A7	A0A3B6KBX5	Pentacotripeptide-repeat region of PRORP domain-containing protein	61.564	1	1	1	1	601	64.6	6.84	0.00
A7	A0A3B5Y562	Polysaccharide biosynthesis domain-containing protein	28.985	1	4	1	1	276	28.4	9.55	0.00
A8	P11383	Ribulose bisphosphate carboxylase large chain	69.182.	4	42	4	1	477	52.8	6.68	34.00
A8	A0A3B6RIQ0	ER lumen protein retaining receptor	20.689	1	1	1	1	290	32.7	9.23	0.00
B1	A0A3B6CFK1	Aminomethyltransferase	30.361	11	40	11	1	415	44.4	8.57	19.22
B1	A0A3B6JLI7	AB hydrolase-1 domain-containing protein	83.333	1	1	1	1	300	32.3	8.21	0.00
B2	A0A3B6CFK1	Aminomethyltransferase	19.518	8	38	8	1	415	44.4	8.57	20.22
B3	A0A3B6LYJ0	Phytocyanin domain-containing protein	4.838	1	2	1	1	124	12.9	9.54	0.00
B4	A0A3B6LWV1	Uncharacterized protein	37.162	4	60	2	1	148	15.6	9.70	26.38
B4	A0A3B6KQS0	Uncharacterized protein	3.445	3	49	1	1	148	15.6	9.70	23.06
B4	A0A3B6N134	Uncharacterized protein	3.445	3	47	1	1	148	15.6	9.70	15.57
B5	A0A3B6QN07	Pentacotripeptide-repeat region of PRORP domain-containing protein	40.384	1	8	1	1	520	58.6	8.95	0.00
B5	A0A3B6IM57	Uncharacterized protein	7.523	1	1	1	1	319	34.6	9.03	0.00
B6	A0A3B6R769	NB-ARC domain-containing protein	0.696	1	2	1	1	1292	145.4	7.44	0.00
B6	A0A3B6EM41	DUF2470 domain-containing protein	22.321	1	1	1	1	448	50.2	5.06	0.00
B7	A0A341ZAA2	Clp R domain-containing protein	17.684	1	4	1	1	622	68.4	7.36	0.00
B7	A0A3B5XYH1	Myosin motor domain-containing protein	0.558	1	2	1	1	1253	142.5	7.96	0.00
B7	A0A3B6NK68	LOB domain-containing protein	33.755	1	2	1	1	237	26.1	6.51	0.00
B7	A0A3B6AX06	DYW domain-containing protein	18.456	1	1	1	1	596	65.9	8.32	0.00
C1	W5AQE7	Genome assembly, chromosome: II	51.204	1	2	1	1	332	34.9	5.90	0.00
C1	A0A3B6MTU7	Uncharacterized protein	13.452	1	2	1	1	669	72.9	8.62	0.00
C1	A0A3B6IK49	NB-ARC domain-containing protein	0.755	1	1	1	1	926	104.8	7.93	0.00
C1	A0A3B6HTM4	DUF4005 domain-containing protein	4.225	1	2	1	1	284	30.5	10.43	0.00
C1	A0A3B5YZA7	FAD-binding domain-containing protein	12.465	1	1	1	1	722	78.8	8.90	0.00
C2	A0A3B6SEK6	Importin N-terminal domain-containing protein	0.965	1	1	1	1	829	92.0	5.52	0.00
C2	A0A3B6GR10	Fungal lipase-like domain-containing protein	11.363	1	1	1	1	528	57.8	7.24	0.00
C2	A0A3B5XYH1	Myosin motor domain-containing protein	0.558	1	1	1	1	1253	142.5	7.96	0.00
C2	A0A1D5UZL0	Genome assembly, chromosome: II	11.976	1	1	1	1	668	72.5	7.84	0.00
C3	A0A3B6G378	NB-ARC domain-containing protein	0.508	1	1	1	1	1179	131.8	7.39	0.00
C3	A0A3B6AZ03	DM2 domain-containing protein	11.406	1	4	1	1	526	57.5	9.66	0.00

C3	A0A3B6ES89	ABC transporter domain-containing protein	0.415	1	1	1	1	1445	162.3	7.17	0.00
C3	A0A3B6JHY7	SAP domain-containing protein	0.813	1	1	1	1	738	80.1	4.86	0.00
C3	A0A3B6ER13	Glycosyltransferase	11.650	1	1	1	1	515	55.3	7.56	0.00
C4	A0A3B6LWV1	Uncharacterized protein	37.162	4	30	2	1	148	15.6	9.70	30.96
C4	A0A3B6KQS0	Uncharacterized protein	37.162	4	23	2	1	148	15.6	9.70	20.50
C4	A0A3B6KRY2	Photosystem I reaction center subunit II, chloroplastic	34.146	1	1	1	1	205	21.9	9.70	0.00
C4	A0A3B6N134	Uncharacterized protein	37.162	4	22	2	1	148	15.6	9.70	17.17
C5	A0A3B6S9S9	MATH domain-containing protein	41.269	1	1	1	1	315	35.0	8.85	0.00
C5	A0A3B6SFH1	Peroxidase	8.169	3	11	1	1	355	37.5	6.77	4.80
C5	A0A3B6N456	NB-ARC domain-containing protein	0.682	1	1	1	1	1025	114.1	8.00	0.00
C5	A0A3B6LUR4	Bifunctional inhibitor/plant lipid transfer protein/seed storage helical domain-containing protein	40.609	1	4	1	1	197	19.6	8.13	0.00
C5	A0A3B6TLZ2	Peroxidase	81.005	3	18	1	1	358	37.7	6.77	14.04

Table S4. Agreement scores (Kappa values) between BP terms in the mutant lines.

**BP terms**	**Cellular process**	**Homeostatic process**	**Metabolic process**	**Response to stimulus**
Cellular process	---			
Homeostatic process	0	---		
Metabolic process	N/A	0	---	
Response to stimulus	0	1	0	---

Table S5. Agreement scores (Kappa values) between BP terms in in all lines.

**BP terms**	Cellular process	Homeostatic process	Metabolic process	Response to stimulus
Cellular process	---			
Homeostatic process	0	---		
Metabolic process	N/A	0	---	
Response to stimulus	0	-0.3125	0	---

Table S6. STIs of each line for several traits.

Trait	Parental	Mutant 4	Mutant 5	Mean	Standard deviation
**Chlorophyll Content**	0.6541	0.7879	0.8158	0.7526	0.0864
**Proline Content**	2.7160	1.5440	2.1880	2.1493	0.5869
**TBARS Content**	1.8945	1.1891	1.3095	1.4644	0.3773
**CAT Activity**	1.9796	1.7817	1.6734	1.8116	0.1552
**POX Activity**	2.5269	1.8729	2.0053	2.1350	0.3457
**SOD Activity**	2.0576	1.6891	1.4995	1.7487	0.2837
**TaCAT Fold Change**	2.5961	2.5157	2.5151	2.5423	0.0466
**TaP5CS Fold Change**	2.5509	2.3594	2.0450	2.3184	0.2554
**TaPOX Fold Change**	1.7733	2.5906	2.6803	2.3481	0.4997
**TaSOD2 Fold Change**	2.4866	2.4201	2.3550	2.4206	0.06581
**Mean**	2.1236	1.8751	1.9087	---	---
**Standard deviation**	0.6156	0.6017	0.5816	---	---

Table S7. Sequence of the primers and the accession number of the corresponding genes and their amplicon size (bp).

Gene	Accession No	Amplicon size	Primer Sequence
TaSOD2	FJ890987.1	225	F: 5′-AGTTCGATTGGCCTGTGGTA-3′
			R: 5′-GATCCCCGAGTCCAGATGAG-3′
TaCAT	D86327.1	292	F: 5′-TCACCTTCCTCTTCGACGAC-3
			R: 5′-AGCACGATGTTGATCCCTTT-3′
TaPOX	X53675.1	263	F: 5′-ACACCAACATCAACACTGCC-3′
			R: 5′-CATCTTGATCATGGCCGTCG-3′
TaP5CS	AY888045.1	157	5'-TACAGTTCTATGGCTTGCACAGT-3'
			R: 5'-ATACGGCAGCACTATCAACTTGA-3'
TaActin	GQ339780.1	208	F: 5′-CGTGTTGGATTCTGGTGATG-3′
			R: 5′-AGCCACATATGCGAGCTTCT-3′
